# Parallel joint encoding for drone-view object detection under low-light conditions

**DOI:** 10.3389/frai.2025.1622100

**Published:** 2025-09-22

**Authors:** Liwen Liu, Bo Zhou, Qiqin Li, Gui Fu, You Wang, Hongyu Chu

**Affiliations:** ^1^Institute of Electronic and Electrical Engineering, Civil Aviation Flight University of China, Guanghan, China; ^2^School of Information Engineering, Southwest University of Science and Technology, Mianyang, China

**Keywords:** drone-view object detection, image enhancement, unmanned aerial vehicle, low-light conditions, parallel neural network

## Abstract

Under low-light conditions, the accuracy of drone-view object detection algorithms is frequently compromised by noise and insufficient illumination. Herein, we propose a parallel neural network that concurrently performs image enhancement and object detection for drone-view object detection in nighttime environments. Our innovative coevolutionary framework establishes bidirectional gradient propagation pathways between network modules, improving the robustness of feature representations through the joint optimization of the photometric correction and detection objectives. The illumination enhancement network employs Zero-DCE++, which adaptively adjusts the brightness distribution without requiring paired training data. In our model, object detection is performed using a lightweight YOLOv5 architecture that exhibits good detection accuracy while maintaining real-time performance. To further optimize feature extraction, we introduce a spatially adaptive feature modulation module and a high- and low-frequency adaptive feature enhancement block. The former dynamically modulates the input features through multiscale feature fusion, enhancing the ability of the model to perceive local and global information. The latter module enhances semantic representation and edge details through the parallel processing of spatial contextual information and feature refinement. Experiments on the two data sets of VisDrone2019 (Night) and Drone Vehicle (Night) show that the proposed method improves 3.13 and 3.1% compared with the traditional YOLOv5 method mAP@0.5:0.95, and improves 6.3 and 2% in mAP@0.5, especially in the extreme low light and high noise environment.

Thus, the proposed parallel model is an efficient and reliable solution for drone-based nighttime visual monitoring.

## 1 Introduction

With the exponential advancement of unmanned aerial vehicles (UAVs), they have been increasingly used for object detection, particularly in nighttime surveillance, disaster rescue, and military reconnaissance (Nguyen et al., 2024; Nguyen et al., 2024; Dao et al., 2025). However, nighttime object detection is challenging because of insufficient illumination, noise interference, and low target–background contrast (Deng et al., 2022; Ni et al., 2024), which severely worsen the performance of traditional detection algorithms.

In previous studies, two strategies have been primarily used for improving nighttime detection performance: (1) improvement of the input quality through image enhancement and preprocessing (2) optimization of the structure of the detection network to enhance feature representation. Nevertheless, these methods are typically realized through serial processing frameworks and suffer from three following limitations: (1) isolated training of enhancement and detection networks without task-oriented feature optimization; (2) over-enhancement potentially introduces artifacts that degrade detection performance; (3) computational redundancy leads to suboptimal real-time performance (Xu et al., 2024).

To address these issues, we devised a parallel fusion neural network (PFNN) consisting of concurrently operating illumination enhancement and detection networks with end-to-end joint optimization. First, we designed a parallel fusion architecture that deeply integrates the Zero-DCE++ illumination enhancement network with the YOLOv5 detection network, with feature co-optimization through shared gradients, which improves mAP@0.5 by 2.6% compared to the traditional serial methods. Second, we employed a spatially adaptive feature modulation (SAFM) module to enhance the ability of the model to perceive local and global information via dynamic multiscale feature fusion, effectively improving target discernibility in low-light conditions. Third, a high- and low-frequency adaptive feature enhancement (HLAFE) block was added to the model to strengthen the semantic representation and edge details through spatial context modeling and feature refinement. In experiments on two nighttime drone-view datasets, the complete model showed a 6.3% higher mAP@0.5:0.95 and a 7.1% higher recall rate than the baselines, particularly excelling in extreme low-light environments.

## 2 Research theory

### 2.1 Nighttime image enhancement

Nighttime image enhancement is a critical step in improving object detection performance under low-light conditions. Although classical methods such as histogram equalization, gamma transform, and the Retinex algorithm can improve the image quality to a certain extent, these methods inherently depend on precise *a priori* knowledge to achieve an accurate fit to the data. However, given that the construction of appropriate and effective *a priori* models for complex and variable lighting environments is a challenging task, this dependence inevitably results in the weak generalization ability of such methods in diverse scenarios. Specifically, because of the complexity and uncertainty of lighting conditions, a universally applicable *a priori* framework is difficult to predefine, which limits the effectiveness and adaptability of these methods to different scenarios. Therefore, the key to enhancing the generalization performance of such methods is the development of more flexible and robust *a priori* modeling strategies that could be adapted to different lighting conditions.

Recently developed deep learning approaches can be divided into supervised and unsupervised methods. In supervised learning, the success of the SENet (Hu et al., 2018) attention module has led to the active research and application of attention-based algorithms. This development has considerably enriched the arsenal of processing techniques for visual tasks and markedly improved the performance of image enhancement models under low-light conditions. The MIRNetv1 (Zamir et al., 2020) and v2 (Zamir et al., 2023) models proposed by Zamir et al. employ a multi-resolution convolutional stream architecture that captures multiscale features while effectively fusing feature information of different levels through information exchange between convolutional streams. A key advantage of these models is their nonlocal attention mechanism, which facilitates adaptive multiscale feature fusion via a selective kernel network, thereby preserving image details. Building upon distribution modeling, a normalized flow framework (Wang et al., 2022) has been developed based on a normalized flow model, providing a robust reference benchmark for low-light image enhancement by simulating the capture of image characteristics under daytime conditions. The self-attentive SNR transformer proposed in (Xu et al., 2022) features a self-attentive machine SNR transformer module that dynamically assesses the contributions of individual pixels based on peak signal-to-noise ratios in various regions of an image, enabling the selective extraction of either local or global information depending on the assessed contribution size.

In supervised learning, training is performed on labeled samples, whereas in unsupervised learning, it is done on unlabeled samples. Jin et al. (2022) highlighted the necessity of balancing the enhancement of low-light areas with overexposure suppression in bright regions because of the complexity of nighttime images. They proposed an innovative unsupervised integration framework that combines layer decomposition with light effect suppression to intelligently optimize the light intensity distribution. However, this unsupervised approach struggles with noise suppression. To address this issue, Xiong et al. (2022) designed a decoupling network containing two GAN subnetworks for the fine decomposition and denoising of images, respectively. This method has shown good noise suppression performance through the use of an adaptive content loss function.

The Zero-DCE series, as a representative unsupervised method, enhances images without requiring paired training data (Nguyen et al., 2024). Thus, herein, Zero-DCE++ was fused in parallel with the object detection network, enabling task-oriented image enhancement through end-to-end joint optimization, thereby overcoming the limitations of conventional serial processing.

### 2.2 Drone-view object detection

Traditional object detection methods usually perform well in scenes with clear visibility but show notably worse performance on nighttime and high-altitude imagery. To address this issue, the joint training of end-to-end image enhancement and object detection networks has been considered.

Liu et al. (2021) introduced the ED-TwinsNet architecture, which seamlessly integrates image enhancement with face detection in a low-light environment through the deep fusion of intermediate feature levels across two subnetworks. Chen et al. (2020) proposed a related but distinct approach: a comprehensive framework that unprecedentedly unifies illumination enhancement and target detection. This framework initially employs a dynamic filter network to generate a set of adaptive convolutional kernels for the fine-grained enhancement of the input. Subsequently, the processed images are fed to an optimized variant of the Fast R-CNN. Notably, in this framework, the weights computed during the enhancement phase are directly applied to the classification loss function of the region proposal network, resulting in substantial improvements in the overall detection performance as well as exceptional flexibility and efficiency.

Wang et al. (2020) adopted a distinct methodology: they developed a hybrid illumination enhancement technology that elegantly integrates the optimal hyperbolic tangent with the enhanced BM3D (Dabov et al., 2007) denoising algorithm. Hao et al. (2021), in contrast, focused on real-time detection and introduced the Low-Light Enhancement Detector, a single-lens real-time target detector tailored for night environments. They bolstered the adaptability of the detection model to such environments through the efficient integration of features and the meticulous adjustment of channel attention mechanisms, substantially improving real-time object detection performance in challenging low-light settings.

Drone-view object detection presents unique challenges, including significant scale variations, complex backgrounds, and perspective distortion. The YOLO algorithms have been widely adopted in UAV applications because of their efficiency and accuracy (Nguyen et al., 2024; Zeng et al., 2023). Thus, herein, we employed lightweight YOLOv5 as the detection backbone of our parallel fusion architecture. Overall, owing to the optimized network structure and training strategy, our model showed enhanced detection accuracy while maintaining real-time performance.

### 2.3 Feature enhancement and modulation

Feature enhancement and modulation are vital for boosting the detection performance. The SAFM enhances the ability of the model to capture local and global information through dynamic multi-scale feature selection. The HLAFE block combines a spatial context module (SCM) and a high- and low-frequency feature extraction module (HLFEM). The SCM employs large-kernel group convolutions to expand the receptive field and strengthen global contextual understanding, whereas the HLFEM accentuates critical edges and structural information through feature sharpening and contrast enhancement (Nguyen et al., 2024). The integration of SAFM and HLAFE enables the model to accurately capture target features in complex nighttime scenarios, thereby equipping the PFNN with robust feature extraction capabilities.

## 3 Experimental methodology

### 3.1 PFNN architecture

**Figure 1:**
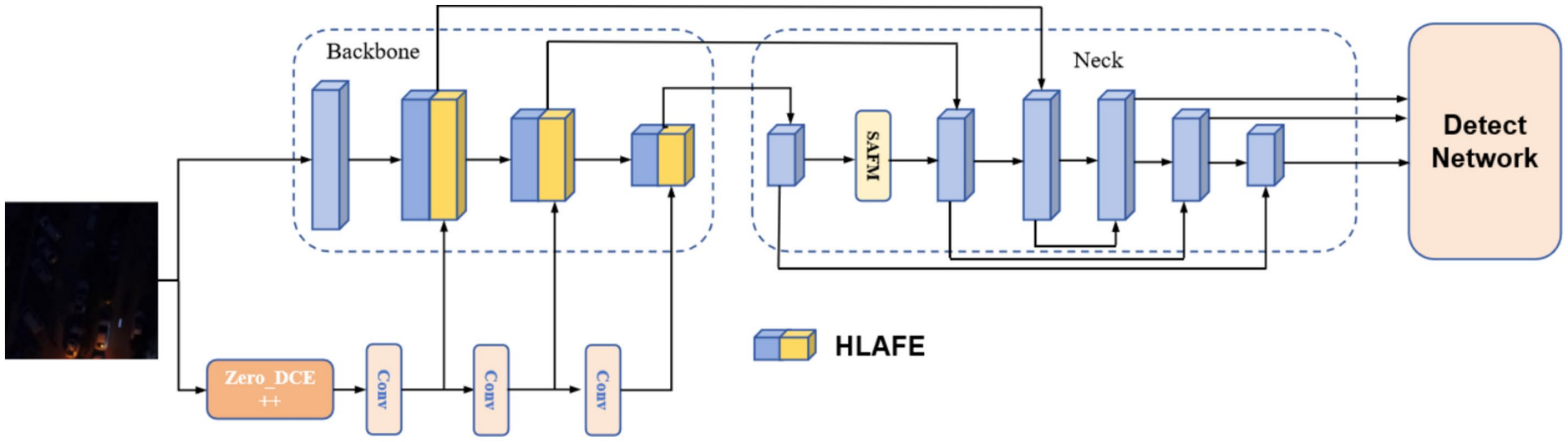
Architecture of the PFNN.

To address the limitations of traditional two-stage networks (enhancement followed by detection), including limited feature correlation and excessive processing latency, we propose a PFNN architecture (Figure 1). This framework integrates an illumination enhancement network with an object detection network through end-to-end joint optimization, enabling cross-feature fusion and adaptive adjustment. The core advantages of our architecture include (1) enhanced computational efficiency with reduced processing delay; (2) stronger feature interactions between subnetworks, allowing the enhancement network to learn detection-favorable representations; and (3) task-oriented feature optimization via gradient sharing, mitigating artifact issues caused by over-enhancement.

In our framework, Zero-DCE++ (Guo et al., 2020) is employed as the illumination enhancement module and lightweight YOLOv5 as the detection module. As shown in Figure 1, the input images are simultaneously processed by Zero-DCE++ to enhance brightness and by the YOLOv5 backbone for feature extraction. Using the zero-reference depth method, Zero-DCE++ adaptively adjusts the luminance distribution through unsupervised learning, considerably improving detection performance in low-light conditions and eliminating the need for paired training data or external supervision (Liu et al., 2024). Parallel processing ensures that enhanced visual features directly participate in detection, enhancing model robustness and accuracy.

### 3.2 SAFM

Noise interference and insufficient illumination in low-light imagery hinder effective feature extraction. The SAFM module was introduced to address this issue (Dao et al., 2025); this module improves feature discriminability through multiscale processing and dynamic modulation.

**Figure 2:**
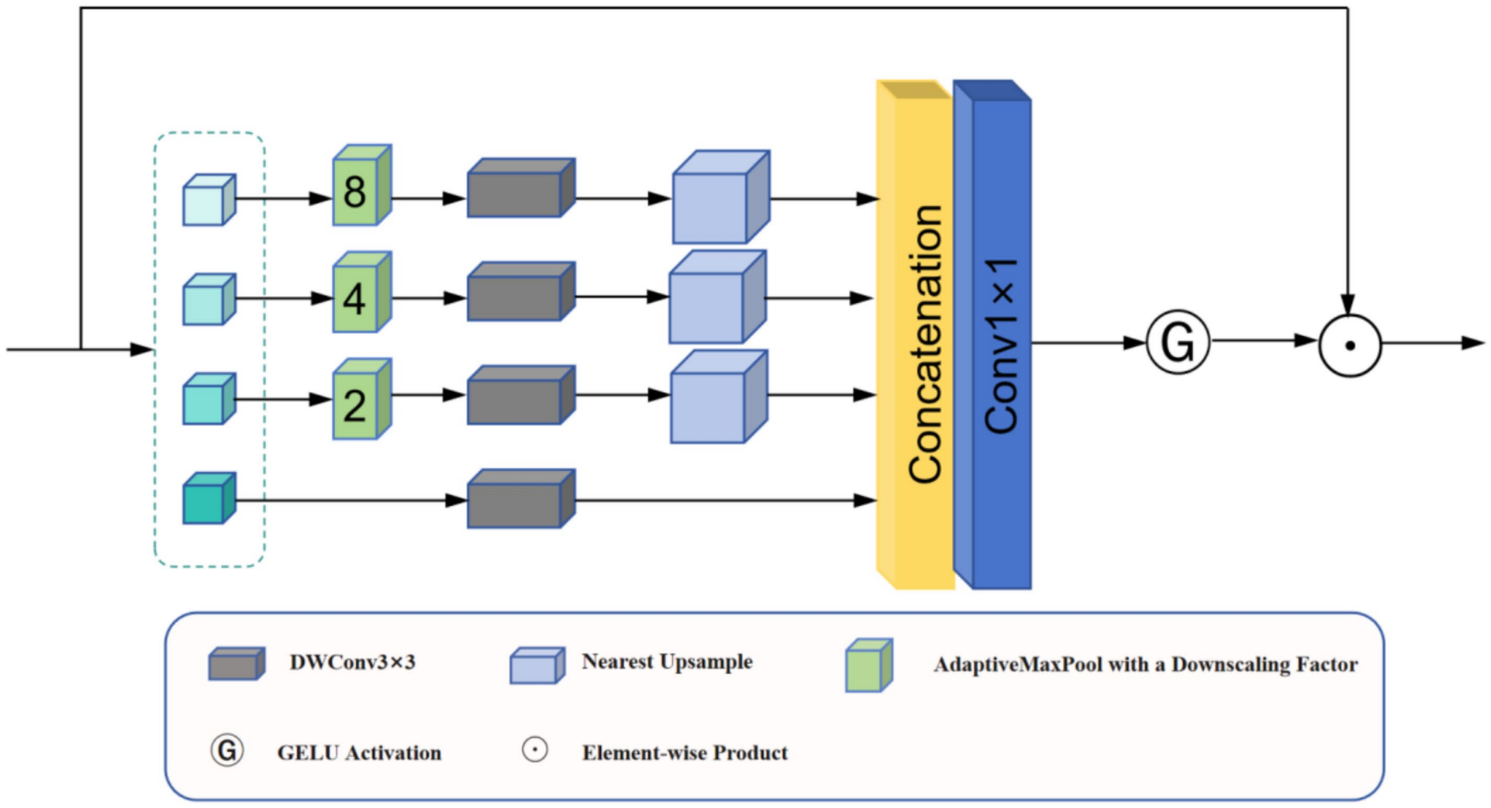
Architecture of the SAFM module.

As shown in Figure 2, the SAFM module first performs channel splitting on the normalized input features ( X ) (Equation 1). The features are divided into four partitions for differential processing. The first partition undergoes depth-wise separable convolution (DW-Conv) for local feature extraction (Equation 2):


(1) $$\hat{X_{0}}=\mathrm{DW}-\mathrm{Con}v_{3\times 3}(X_{0})$$

(2) $$\left[X_{0},X_{1},X_{2},X_{3}]=\text{Split}(X\right)$$

The remaining partitions are processed through multiscale operations (down-sampling, convolution, and up-sampling) (Equation 3):


(3) $$\hat{X_{i}}={↑}_{p}(\mathrm{DW}-{\text{Conv}}_{3\times 3}({↓}_{\frac{p}{2^{i}}}(X_{i}))),1\leq i\leq 3$$

The obtained multiscale features are aggregated via max-pooling and 1×1 convolution (Equation 4):


(4) $$\hat{X}={\text{Conv}}_{1\times 1}(\text{Concat}([{\hat{X}}_{0},{\hat{X}}_{1},{\hat{X}}_{2},{\hat{X}}_{3}]))$$

The integrated features undergo GELU activation (Cao et al., 2021) to generate attention maps for dynamic feature weighting (Equations 5, 6):


(5) $$\hat{X}=\text{GELU}(\hat{X})$$

(6) $$\bar{X}=\phi (\hat{X})⊙X$$

This mechanism enables the automatic selection of discriminative features across multiple scales, considerably enhancing the detection robustness in low-light conditions. The experimental results indicate that SAFM improves detection precision in complex nighttime environments while maintaining computational efficiency, benefiting the autonomous navigation and environmental perception of UAVs.

### 3.3 HLAFE block

**Figure 3:**
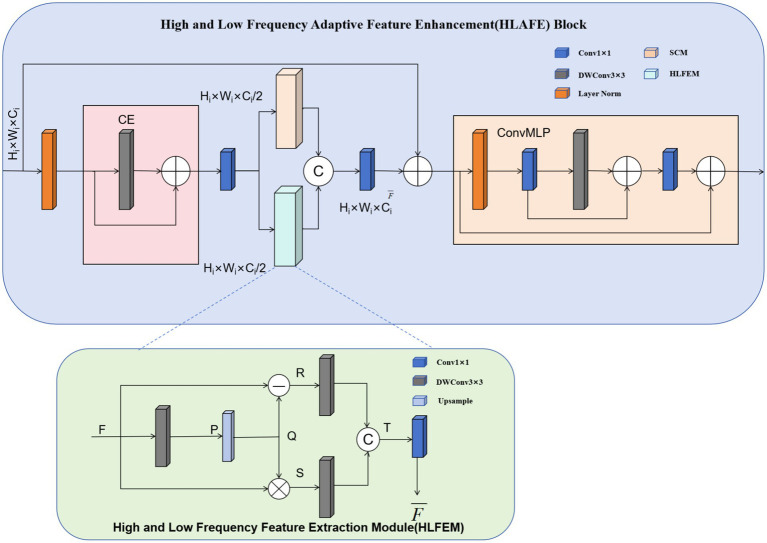
Architecture of the HLAFE module.

The HLAFE block was employed to optimize feature representations and improve the capability of the model to identify critical targets and thereby address challenges such as scale variations and background interference. As shown in Figure 3, HLAFE employs multi module collaboration to enhance features, enabling the comprehensive learning of global semantics and local details.

The HLAFE module operates through coordinated processing by the SCM (Hu et al., 2018) and HLFEM, enhancing features via two parallel pathways to capture rich contextual information and fine-grained semantic features (Nie et al., 2019; Shi et al., 2016).

### 3.4 HLFEM design

The design of the HLFEM module draws inspiration from image sharpening and contrast enhancement techniques. In image sharpening, high-frequency information is accentuated to improve image clarity, whereas in contrast enhancement, the contrast of low-frequency information is increased to improve the overall structural perception. Inspired by this, the HLFEM acquires low-frequency information through down-sampling and smoothing while extracting high-frequency details by computing residuals between the original features and low-frequency information. Subsequently, different frequency information is integrated to enhance both the local details and the semantics of the features, thereby optimizing the segmentation performance.

### 3.5 HLAFE module architecture

The HLAFE module consists of a convolutional embedding (CE) module, an SCM, an HLFEM, and a convolutional multilayer perceptron (ConvMLP). The input features are first processed by the Layer Norm and 1 × 1 convolution layer (CE), which compresses the number of channels to half of the original dimension. This reduces the computational cost while promoting feature mixing. The compressed features are subsequently channeled into the SCM consisting of grouped convolutions with expansive kernel configurations (kernel size of 7 × 7). Concurrently, the compressed feature vectors derived from the CE layer are fed to the HLFEM for progressive feature optimization through attention-guided recalibration. The concatenated outputs from the SCM and HLFEM undergo dimensional projection via a 1 × 1 convolutional layer coupled with a ConvMLP, synergistically enhancing the discriminative feature representations for downstream tasks.

### 3.6 Module implementation details

The SCM employs 7 × 7 group convolutions to enlarge the receptive field and enhance global contextual features. This large-receptive-field design effectively adapts to scale variations in complex scenes, allowing the model to comprehensively discern targets at different scales. At the same time, the HLFEM applies depth-wise convolutional layers to down-sample and smooth features for low-frequency information extraction while simultaneously computing the residuals between the original features and the low-frequency information to extract the high-frequency details. These different frequency features are concatenated and further fused through a projection layer, improving the overall feature representation.

### 3.7 Feature integration

Features processed by SCM and HLFEM are concatenated and fused via 1 × 1 convolution to integrate multiscale and multifrequency information. The fused information is then input into ConvMLP to further improve feature representation. The ConvMLP enhances the nonlinear expressive capabilities of the model through multilayer perception, enabling more effective learning of semantic information in complex scenes, thereby boosting the accuracy and robustness of segmentation.

## 4 Experiments and results

### 4.1 Datasets and experimental setup

Based on prior research, we selected two representative nighttime datasets: VisDrone2019 (Night) and Drone Vehicle (Night), for training and evaluation.

The original VisDrone2019 datasets (Hendrycks and Gimpel, 2016) contains UAV-captured video sequences of 10 object categories under daytime and low-light conditions. To construct the nighttime subset, we extracted 2,023 training images and 56 test images from the original data. This subset covers 10 categories, including pedestrians, bicycles, and cars, with an image resolution of 2000 × 1,500 pixels and a minimum target size of 16 × 16 pixels. The scenarios include urban streets and intersections.

The Drone Vehicle datasets contains 56,878 paired RGB and infrared images. To assemble the nighttime subset, we selected 11,406 training and 880 test RGB images captured at night with ground-truth annotations. These 640 × 512 pixel images contain five vehicle categories (e.g., buses, trucks) with substantial illumination unevenness.

The experiments were implemented in PyTorch and run on a PC with an NVIDIA GTX 3090Ti GPU, CUDA 11.0, and CUDNN 8.0. The training hyper parameters include:

Optimizer: Adam with an initial learning rate of 0.015 and a momentum of 0.937.

Batch size: 8 (prevents memory overflow).

Epochs: 300 with Mosaic data augmentation.

The YOLOv5 detector was initialized with pretrained weights, and its parameters were frozen during the initial training stages to preserve the baseline detection capability. In the first 100 epochs, the Zero-DCE++ enhancement network remained frozen to stabilize feature learning. After epoch 100, both networks were jointly optimized end-to-end, enabling gradual coordination between the enhancement and detection modules.

### 4.2 Evaluation metrics

We assessed model performance using the following standard evaluation criteria:

Average Precision (AP): Measures the detection capability for individual categories by balancing precision and recall, the detailed calculation method is shown in Equation 7:


(7) $$\mathrm{AP}=\frac{\mathrm{TP}+\mathrm{TN}}{\mathrm{TP}+\mathrm{TN}+\mathrm{FP}}$$

Recall Rate (R), it evaluates the model’s capacity to capture all positive samples, representing the proportion of avoided false negatives, the detailed calculation method is shown in Equation 8:


(8) $$R=\frac{\mathrm{TP}}{\mathrm{TP}+\mathrm{FN}}$$

Precision Rate (P), it measures the proportion of samples predicted as positive that are actually positive, reflecting the model’s ability to avoid false positives, the detailed calculation method is shown in Equation 9:


(9) $$P=\frac{\mathrm{TP}}{\mathrm{TP}+\mathrm{FP}}$$

where TP is true positives, TNis true negatives,FN is false negatives, and FP is false positives.

Mean AP (mAP): Evaluates the overall classification and localization performance across all categories, the detailed calculation method is shown in Equation 10:


(10) $$\mathrm{mAP}=\frac{1}{n}\sum_{i=1}^{n}{\mathrm{AP}}_{i}$$

where n is the number of categories and ${\mathrm{AP}}_{i}$ is the AP for the $i^{\mathrm{th}}$ category.

### 4.3 Experimental analysis

A series of comparative experiments and ablation studies were conducted to determine the contributions of each module in our model to the overall object detection performance. The experiments were performed on VisDrone2019(Night), Drone Vehicle(Night) and ExDark datasets. The training period in each experiment was 300. In the baseline comparison experiments, the original YOLOv5 model (YOLOv5n) was trained for 300 epochs. As shown in Table 1, our parallel algorithm exhibits the best detection performance. Compared with the YOLOv5 algorithm, our model shows the mAP@0.5 and mAP@0.5:0.95 higher by 3.26 and 4.87%, respectively. Furthermore, compared to the two-stage networks (LIME + YOLOv5, ZeroDCE + YOLOv5, ENGAN + YOLOv5, and RUAS + YOLOv5), our single-stage network exhibits mAP@0.5:0.95 higher by 3.82, 2.07, 1.78, and 1.56%, and mAP@0.5 higher by 2.17, 1.20, 0.60, and 2.89%, respectively. Our model shows higher mAP@0.5:0.95 and mAP@0.5 even than the YOLOv6 and YOLOv7 networks combined with RUAS and ENGAN.

**Table 1:** Performance of different object detection algorithms on ExDark.

Ablation	mAP@0.5	mAP@0.5:0.95	F1-Score
YOLOv5	0.6845	0.4020	0.705
LIME + YOLOv5	0.6954	0.4125	0.713
ZeroDCE + YOLOv5	0.7051	0.4295	0.728
ENGAN + YOLOv5	0.7111	0.4329	0.735
ENGAN + YOLOv7	0.7142	0.4494	0.758
RUAS + YOLOv5	0.6982	0.4351	0.719
RUAS + YOLOv6	0.7101	0.4454	0.752
Ours	0.7171	0.4507	0.772

The results of the comparative experiments indicate that our parallel architecture, integrating an illumination enhancement network with an object detection network (end-to-end joint training framework), outperforms traditional two-stage serial paradigms (independent enhancement followed by detection). Our model achieves an average mAP@0.5 improvement of 1.7% over the existing models in the extreme low-light scenarios. The core mechanism of the proposed approach is the collaborative optimization of the dual-network parameters, enabling the enhancement module to dynamically adapt to the detection task. This eliminates edge artifacts caused by over-enhancement in serial modes and mitigates interference from illumination compensation on target geometric features via gradient back propagation through the shared intermediate layer (Wang et al., 2023). These findings provide critical theoretical support for designing real-time vision systems for dynamic environments such as UAVs for nighttime inspections.

Next, we introduced the SAFM module into the Neck part of the YOLOv5 model. SAFM effectively enhances the capability of the model to learn local and global information through multiscale feature processing. The SAFM module further improves the recovery of details in the images via dynamic feature adjustment, particularly under low-light conditions. Subsequently, the HLAFE module was independently incorporated into the YOLOv5 parallel architecture. The HLAFE module integrates the SCM with the HLFEM to enhance feature representation through parallel processing. This module captures richer contextual information and semantic cues, substantially boosting the detection accuracy.

Building on these results, we combined the HLAFE and SAFM modules in the final model. In this configuration, HLAFE enhances features while SAFM modulates them to improve recognition in complex scenes. After 300 training epochs, the final model shows 40.6 GFLOPs on the VisDrone2019(Night) datasets, with considerably improved detection accuracy. Ablation experiments were performed to systematically validate the contributions of each component of the PFNN to the overall performance in Table 2. The results indicate that the parallel architecture (YOLOv5 + Zero-DCE++) shows a 2.3% higher mAP@0.5 than the serial-structured YOLOv5 + Zero-DCE (mAP@0.5 = 0.178). These results confirm that the proposed parallel design preserves more effective features from enhanced images, thereby overcoming feature degradation observed in serial structures. The YOLOv5 parallel + SAFM model exhibits a mAP@0.5:0.95 of 0.116 (2.06% improvement over the baseline parallel framework), and an R of 0.236. This indicates that SAFM considerably enhances the perception of blurred targets in nighttime scenes through dynamic feature selection (Li et al., 2025).

**Table 2:** Results of the ablation study on VisDrone2019 (night).

Ablation	P	R	mAP@0.5	mAP@0.5:0.95	F1-Score
YOLOv5	0.408	0.183	0.175	0.0837	0.253
YOLOv5(parallel)	0.494	0.198	0.201	0.0954	0.283
YOLOv5(parallel) + SAFM	0.506	0.236	0.233	0.116	0.322
YOLOv5(parallel) + HLAFE	0.495	0.208	0.209	0.0992	0.293
YOLOv5(parallel) + HLAFE + SAFM	0.493	0.254	0.238	0.115	0.335

The complete YOLOv5 parallel + HLAFE + SAFM model shows optimal balanced performance, with mAP@0.5:0.95 reaching 0.115, a 3.13% improvement over the initial YOLOv5 baseline. The full model maintains the high precision of the base architecture while exhibiting a 7.1% higher R owing to the synergistic interaction between the two key modules, validating the effectiveness of the dual feature optimization mechanism under parallel processing.

On the Drone Vehicle(Night) datasets, YOLOv5 parallel + SAFM network shows a mAP@0.5 of 0.738 and an R of 0.692. Notably, the R is higher by 7.2% than that of the baseline (original 0.580), preliminarily validating the effectiveness of the feature modulation strategy in reducing missed detections. For buses—the most stable category in complex urban scenarios—mAP@0.5 reaches 0.916 and mAP@0.5:0.95 reaches 0.618, substantially higher than those for other traffic objects. This improvement correlates with the dynamic adjustment capabilities of SAFM.

For cars, the YOLOv5 (Parallel) + HLAFE architecture shows a mAP@0.5 of 0.902 but an R of 0.66, indicating a potential trade off between the localization precision and the target search capability. The full model (YOLOv5 (Parallel) + HLAFE + SAFM) exhibits an R of 0.697 and a mAP@0.5 of 0.749 owing to coordinated optimization. The spatial weight allocation of SAFM improves truck detection performance mAP@0.5 from 0.528 (SAFM-only) to 0.563, whereas the channel attention of HLAFE maintains car detection precision at 0.902. The complementarity of these two mechanisms mitigates their individual limitations, verifying the cascaded enhancement in the parallel frameworks (Table 3).

**Table 3:** Results of the ablation study on Drone Vehicle (night).

Ablation	P	R	mAP@0.5	mAP@0.5:0.95	F1-SCORE
YOLOv5	0.79	0.63	0.729	0.432	70.09
Yolov5(PARALLEL)	0.821	0.63	0.729	0.447	71.29
YOLOv5(parallel) + SAFM	0.757	0.692	0.738	0.465	72.30
YOLOv5(parallel) + HLAFE	0.796	0.66	0.736	0.46	72.16
YOLOv5(parallel) + HLAFE + SAFM	0.773	0.697	0.749	0.463	73.30

**Figure 4:**
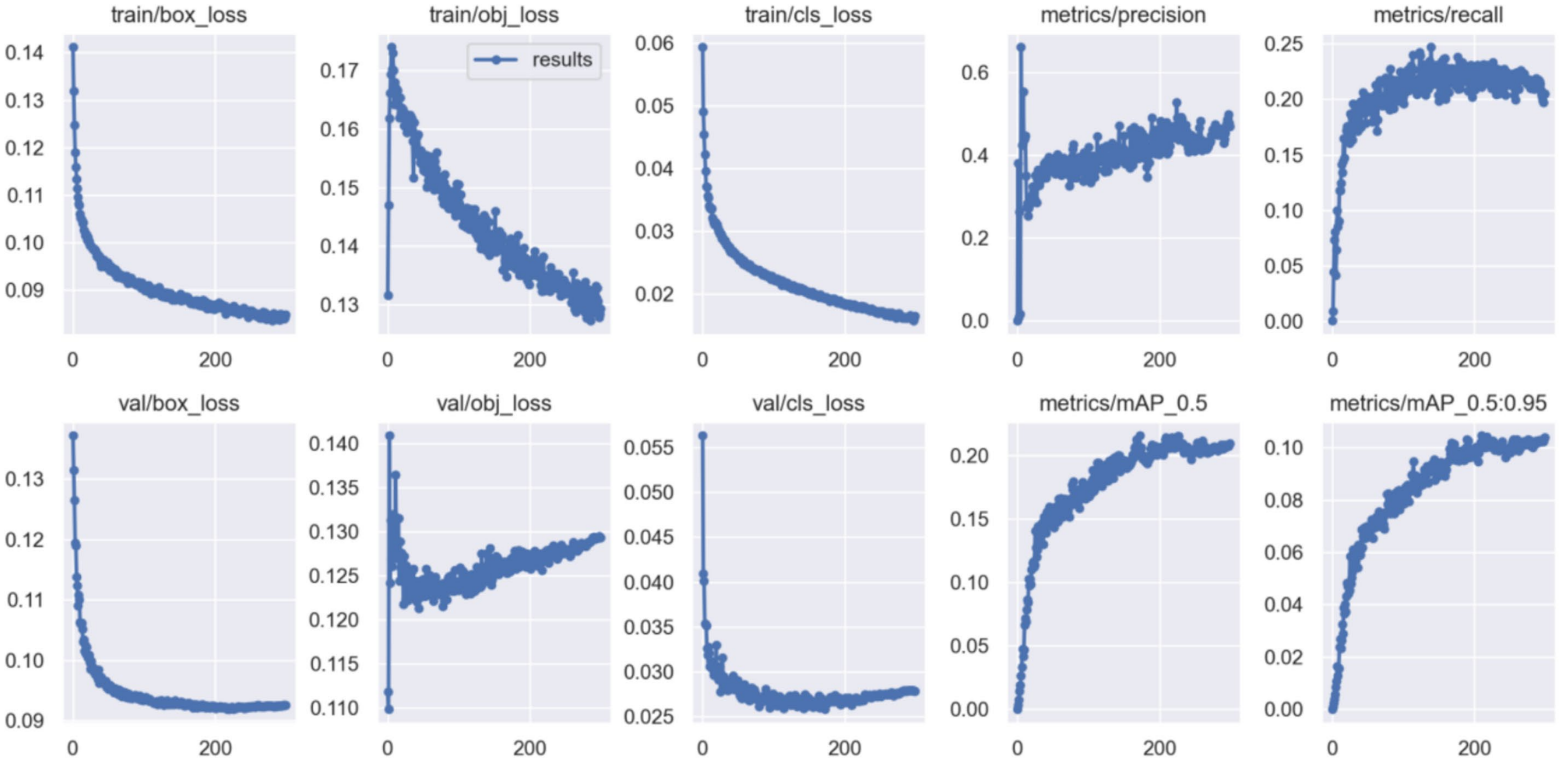
Metric evolution curves.

To address low-light interference and feature representation insufficiency in nighttime detection, our PFNN framework integrates image enhancement and feature optimization. The experimental results indicate the superior performance of our model on both the VisDrone2019(Night) and Drone Vehicle(Night) datasets. Comparative analysis reveals that the Zero-DCE++ & YOLOv5 parallel structure improves mAP@0.5:0.95 by 1.37% (from 0.0827 to 0.0945), highlighting its advantages in feature preservation and joint optimization. The synchronized feature extraction between the enhancement and detection networks prevents the loss of information inherent in the traditional two-stage approaches. Training convergence trends (Figure 4) further confirm stable improvements in key metrics such as R and mAP owing to multitask joint training.

**Figure 5:**
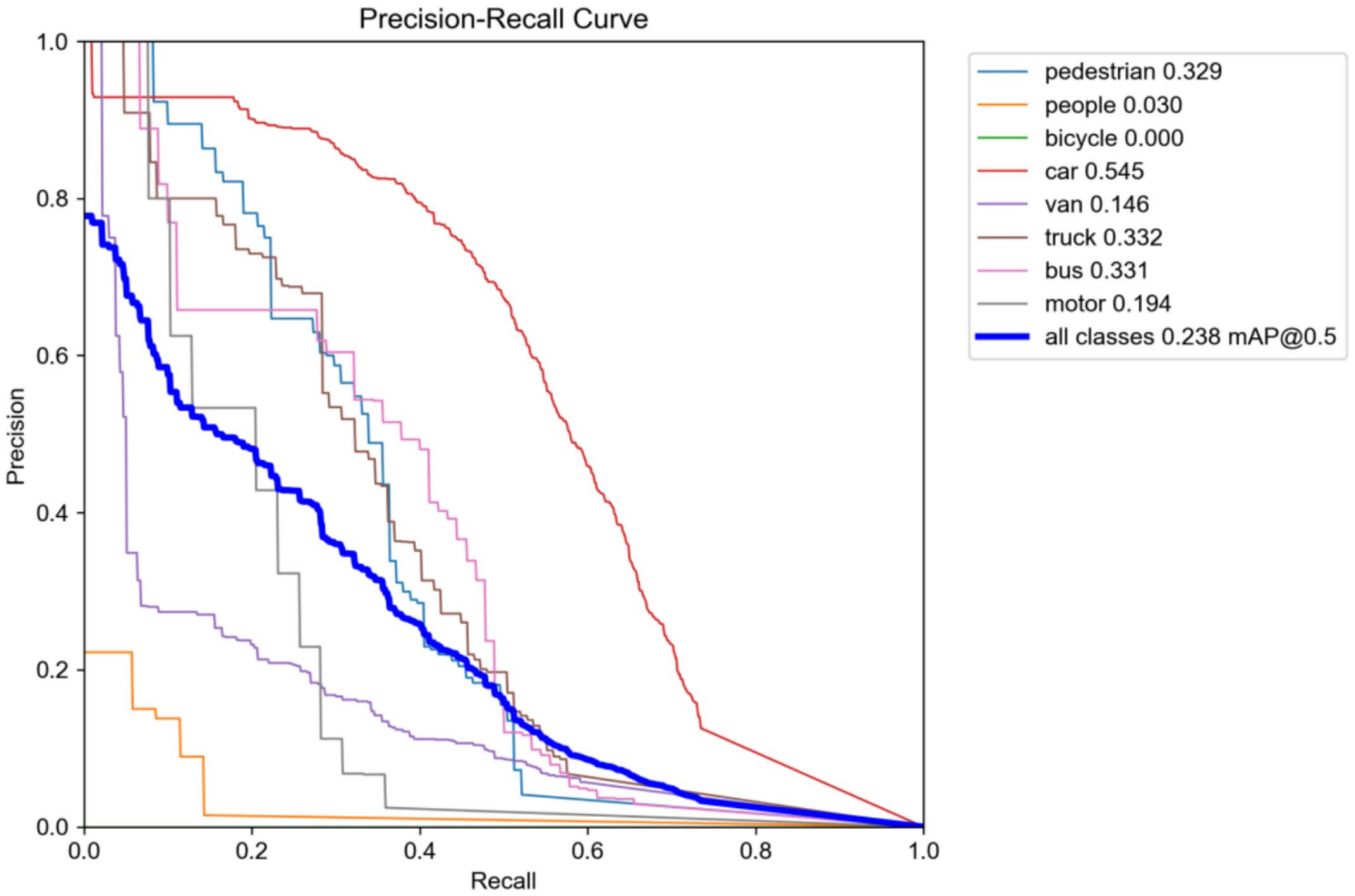
Precision–recall curves on VisDrone2019 (Night).

The SAFM module improves the R by 5.3% (from 0.183 to 0.236) over the baseline owing to its multiscale dynamic feature fusion, substantially improving the perception of blurred targets. The HLAFE block boosts the detection precision for fine-grained objects via spatial context modeling and feature sharpening. On the VisDrone2019(Night) datasets, the highest mAP@0.5 (0.545) is observed for the car category, representing an 8.2% improvement over the baseline, as evidenced by classification accuracy gains in the precision–recall curves (Figure 5).

**Figure 6:**
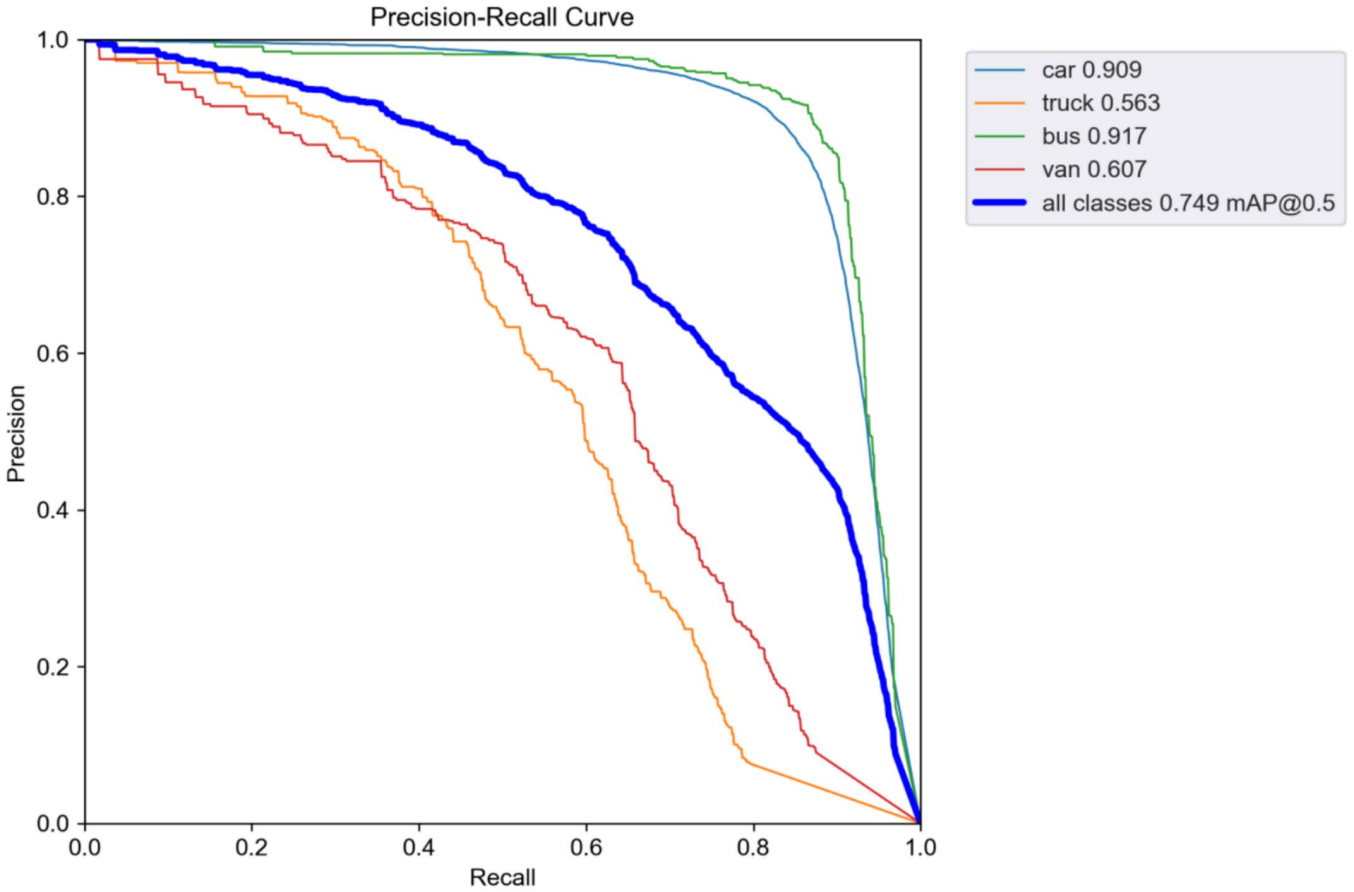
Precision–recall curves on Drone Vehicle (Night).

The integrated HLAFE + SAFM model shows a mAP@0.5 of 0.749 (2.5% improvement over YOLOv5) and an R of 0.697 on Drone Vehicle(Night), with mAP@0.5 for the truck category reaching 0.563 (4.3% baseline gain). These results confirm the synergistic effects of the dual feature optimization in complex nighttime scenarios, further validated by improvements observed in the precision–recall curve (Figure 6).

**Figure 7:**
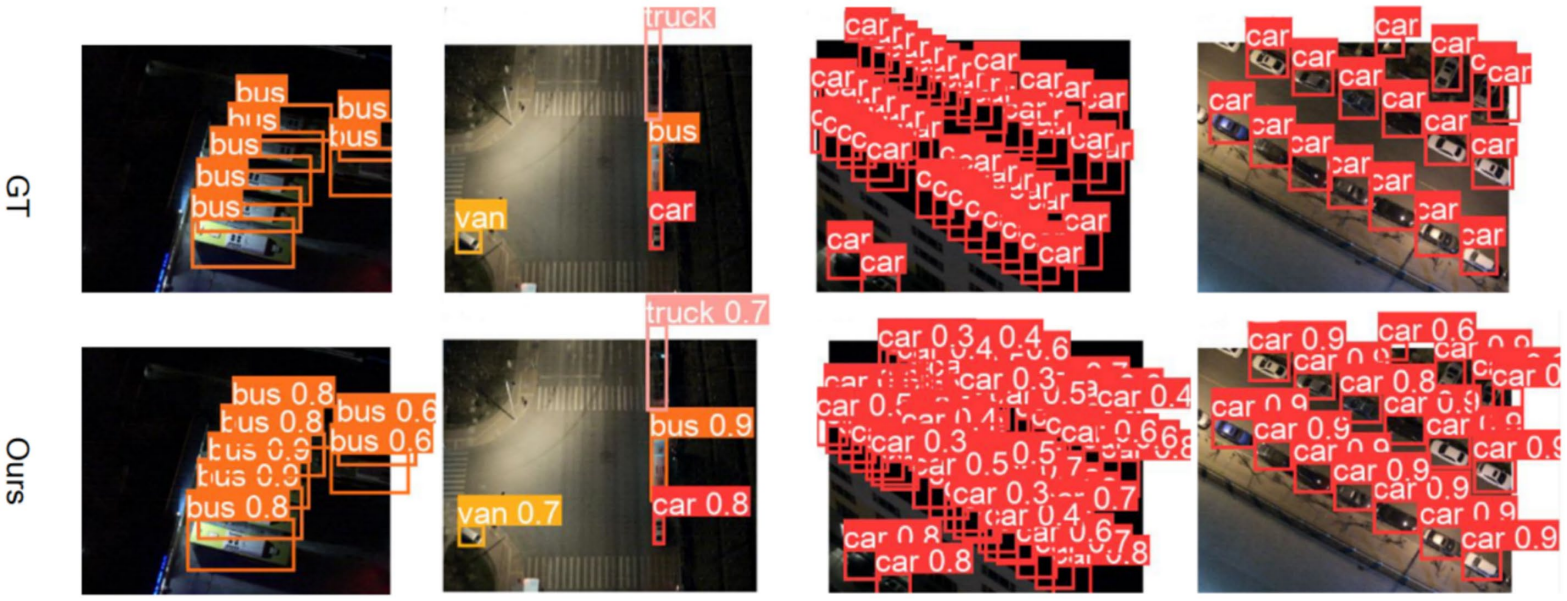
Visual comparison of the detection results.

Thus, owing to the dual feature optimization mechanism, our model exhibits excellent performance in practical nighttime detection. The comparative detection results in Figure 7 visually validate improved boundary localization accuracy for vehicles and reduced false positives for pedestrians.

## 5 Conclusion

Here, we propose an innovative PFNN to address challenges in UAV nighttime object detection such as low illumination and high noise. In our method, illumination enhancement and target detection are synergistically optimized through a jointly optimized dual-branch architecture. Specifically, the unsupervised Zero-DCE++ enhancement module performs adaptive luminance correction, effectively eliminating the dependency on paired training data inherent in conventional methods. At the same time, the improved lightweight YOLOv5 detection network substantially improves feature representation in complex scenarios via SAFM and HLAFE. The SAFM module enhances the local–global feature perception through multiscale feature fusion, and HLAFE preserves the target edge details via parallel context modeling and feature refinement.

In the experiments on the VisDrone2019(Night) and Drone Vehicle(Night) datasets, our model showed 6.3% higher mAP@0.5:0.95 and 7.1% higher R than the baseline under extremely low illumination conditions. This work not only provides a reliable algorithm for drone-view nighttime visual monitoring but also offers new research perspectives for multi-modal sensor fusion (e.g., infrared/visible-light coordination) through the proposed feature modulation mechanisms and parallel optimization framework. Future research should focus on cross modal feature alignment strategies and dynamic resource allocation mechanisms to further enhance system robustness in complex illumination environments (Wei and Du, 2023; Lian et al., 2023).

## Data Availability

The raw data supporting the conclusions of this article will be made available by the authors, without undue reservation.
